# The Stoop-Squat-Index: a simple but powerful measure for quantifying whole-body lifting behavior

**DOI:** 10.1186/s40945-022-00135-4

**Published:** 2022-04-22

**Authors:** Stefan Schmid

**Affiliations:** 1 Bern University of Applied Sciences, Department of Health Professions, Division of Physiotherapy, Spinal Movement Biomechanics Group, Murtenstrasse 10, 3008 Bern, Switzerland; 2 University of Basel, Faculty of Medicine, Klingelbergstrasse 61, 4056 Basel, Switzerland

**Keywords:** Lifting strategy, Lifting technique, Object lifting, Kinematics

## Abstract

**Background:**

Most of the studies evaluating lifting behavior only focus on very localized parameters such as lumbar spine flexion, while evaluations of whole-body strategies are largely lacking. To enable relatively simple evaluations of whole-body strategies, this study aimed at developing a novel index for quantifying the stoop-squat behavior, and to establish normative values of the index for healthy pain-free adults.

**Methods:**

A novel index, the Stoop-Squat-Index, was developed, which describes the proportion between trunk forward lean and lower extremity joint flexion, with possible values ranging from 0 (full squat lifting) to 100 (full stoop lifting). To enable the interpretation of the index in a real-life setting, normative values for lifting a moderately-weighted object (15-kg-box) with a full squat and a full stoop technique were established using motion capture data from 30 healthy pain-free individuals that underwent motion analysis of squat and stoop lifting in the context of a previously conducted study.

**Results:**

The results showed mean index values of lower than 30 and higher than 90 for the most relevant phases of the squat and stoop movements, respectively, with mean index values differing significantly from each other for the full duration of the lifting phases.

**Conclusions:**

The main advantages of the index are that it is simple to calculate and can not only be derived from motion capture data but also from conventional video recordings, which enables large-scale in-field measurements with relatively low expenditure. When used in combination with lumbar spine flexion measurements, the index can contribute important information, which is necessary for comprehensively evaluating whole-body lifting strategies and to shed more light on the debate over the connection between lifting posture and back complaints.

## Background

Most health care professionals and manual material handling advisors as well as guidelines issued by occupational safety organizations and even national health institutes such as the North American NIH or the British NHS promote the so called squat lifting technique as the “correct” and safe way compared to its opposite the stoop lifting technique, which is considered dangerous for the back and therefore strongly advised against [1–4]. The squat lifting technique is thereby defined as flexing the knees and keeping the back as straight as possible (i.e., no forward flexion in the spine), while the stoop lifting technique is mainly achieved by a forward flexion of the spine without bending the knees.

However, despite these widely accepted guidelines, there is no consistent evidence which supports advocating squat over stoop lifting to prevent back injury. While some earlier observational evidence showed positive correlations between trunk forward lean and low back pain (LBP) incidence in occupational settings [5], a recent meta-analysis revealed that greater lumbar spine flexion during lifting was neither a risk factor for LBP onset/persistence, nor a differentiator of individuals with and without LBP [6]. It should be noted, however, that the findings of this meta-analysis were only based on low quality evidence, which is why more high-quality studies are needed before definitive conclusions can be made. In this regard, all the observational and within the meta-analysis presented biomechanical studies only focused on partial aspects of lifting such as anterior trunk lean or lumbar spine flexion, and did not consider evaluations of whole-body lifting strategies, which might be equally important to shed more conclusive light on the debate over the connection between lifting posture and back complaints.

Such evaluations could be implemented for example by using laboratory-based three-dimensional optical motion capture techniques, which however would obviously not be suitable when aiming at large-scale measurements in occupational settings. A possible way for more easily determining whole-body movement strategies would be through an adequate index that could be derived for example from conventional video recordings. Currently available indices in the context of object lifting such as the well-known NIOSH Lifting Equation [7], however, do not consider any motion-related parameters that would allow an appropriate evaluation of movement strategies and are therefore not suitable for this purpose.

For these reasons, this study aimed at developing a novel index for quantifying the stoop-squat behavior, and to establish normative values of the index for lifting a moderately-weighted object with a full squat and a full stoop technique in healthy pain-free adults.

## Methods

### Development of the stoop-squat-index

To quantify whole-body strategies during object lifting, the Stoop-Squat-Index (*StSq*) was developed, which describes the proportion between trunk forward lean and lower extremity joint flexion based on the formula:

1.1 $$ S\mathrm{t} Sq=100-\left(\frac{\left( Vert\_{HJC}_{Standing}- Vert\_{HJC}_{Bending}\right)\ast 100}{Vert\_C{7}_{Standing}- Vert\_C{7}_{Bending}}\right) $$

with the boundary conditions:

1.2 $$ Vert\_C{7}_{Standing}> Vert\_C{7}_{Bending} $$

1.3 $$ Ver t\_{HJC}_{Standing}\ge Ver{t_{HJC}}_{Bending} $$

1.4 $$ Ver t\_{HJC}_{Standing}- Ver{t_{HJC}}_{Bending}\le Ver{t_{C7}}_{Standing}- Ver t\_C{7}_{Bending} $$

**Fig. 1:**
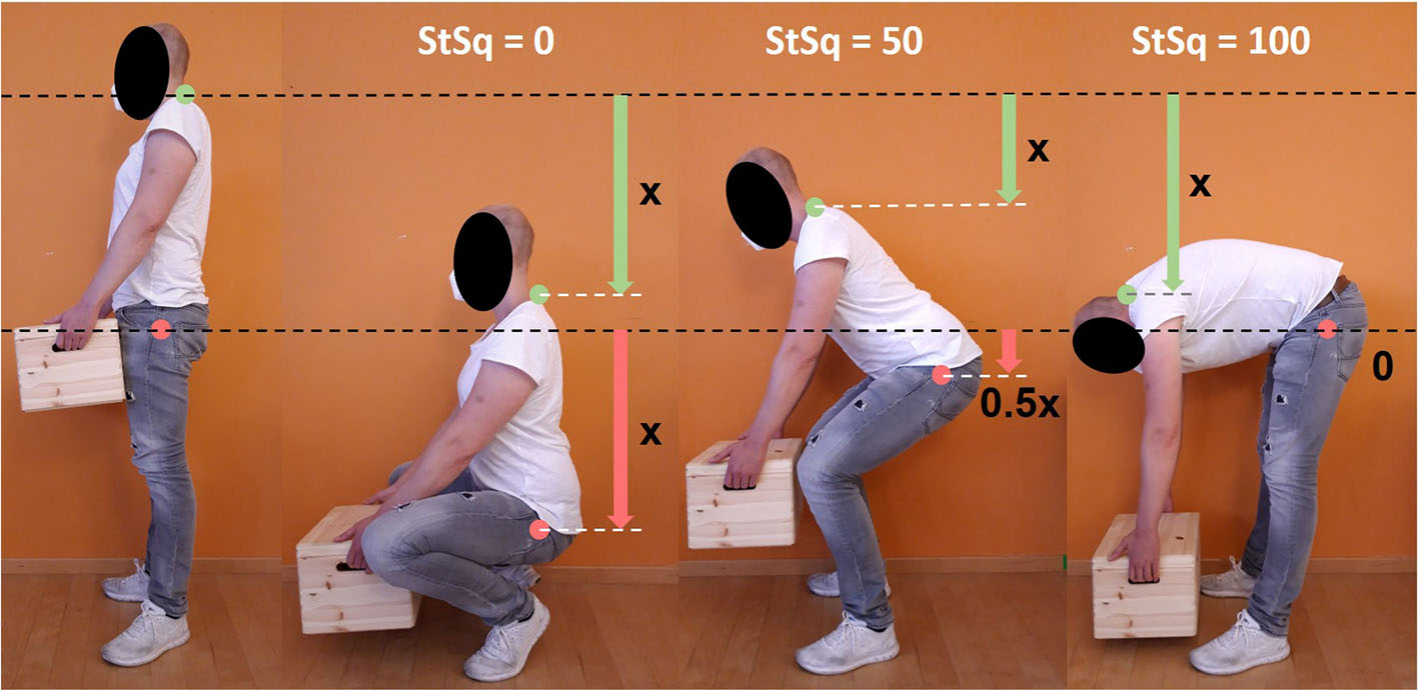
Interpretation of the Stoop-Squat-Index. An index of 0 indicates a full squat movement and an index of 100 a full stoop movement. Values in between indicate partially squat and partially stoop movements

The variables *Vert_HJC* and *Vert_C7* represent the vertical positions of the hip joint center as well as the tip of the C7 spinous process, respectively, during standing and bending. An index of 0 thereby indicates a full squat movement, represented by a C7 downward displacement caused entirely by lower extremity joint flexion, whereas an index of 100 indicates a full stoop movement, represented by a C7 downward displacement caused entirely by trunk forward lean (Fig. 1). Any value in between, e.g., an index of 50, indicates a lifting movement that is neither full squat nor full stoop, represented by a C7 downward displacement caused partially by lower extremity joint flexion and partially by trunk forward lean.

**Fig. 2:**
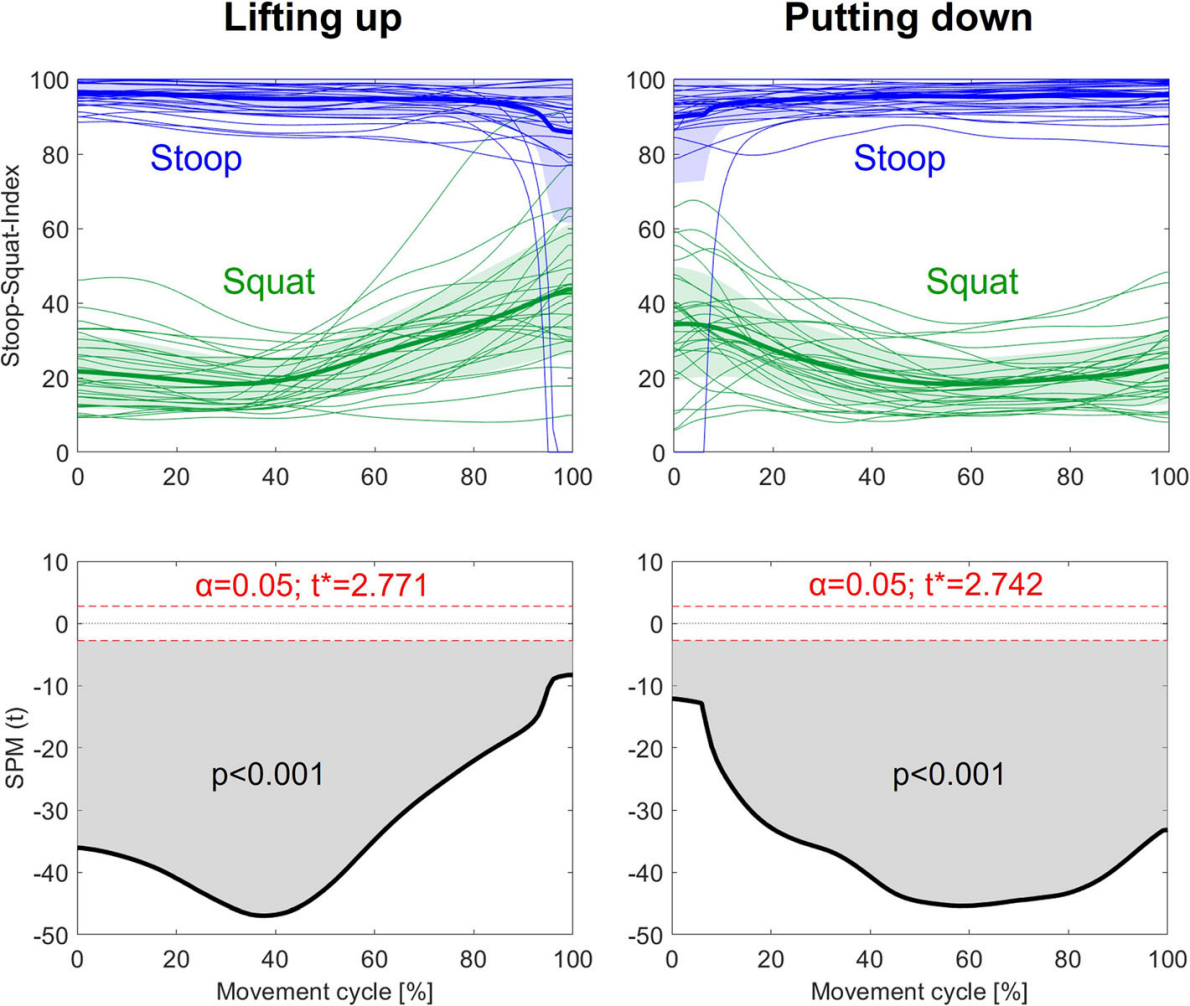
Top row: Normative values of the Stoop-Squat-Index for lifting up and putting down a 15 kg-box based on a sample of 30 healthy pain-free adults, with the thick lines and shaded areas representing mean values und standard deviations. Bottom row: Comparison of the Stoop-Squat-Indices between the two lifting techniques using Statistical Parametric Mapping-based paired samples t-tests

### Establishing normative values

To enable the interpretation of the Stoop-Squat-Index in a real-life setting, normative values were established using pre-processed motion capture data (i.e., labeled and filtered three-dimensional trajectories of 58 retro-reflective skin markers recorded with a 16-camera Vicon motion capture system) from a sample of 30 healthy pain-free individuals that underwent motion analysis during lifting a moderately-weighted object (15-kg-box) with a full squat and a full stoop lifting technique in the context of a previously conducted and published study [8]. This sample consisted of 20 males and 10 females with a mean age of 31.8 ± 8.5 years and a mean BMI of 23.3 ± 2.4 kg/m^2^, which were recruited by flyer from the personal and workplace environment of the investigators of this previous study. They asked the participants to perform five repetitions of each lifting up and putting down a 15 kg-box using first a squat and then a stoop lifting technique. For squat lifting, participants were thereby instructed to lift the box with the back kept as straight as possible with mainly flexing the knees, and for stoop lifting, to lift the box by bending forward with a clear flexion in the spine and with the knees kept as straight as possible. Using these pre-processed motion capture data as well as information on the participants’ body height and leg length from the previous study, lifting up and putting down phases of the squat and stoop lifting maneuvers were identified using a MATLAB-based event detection algorithm (R2020a, MathWorks Inc., Natrick, MA, USA) [9]. To calculate the Stoop-Squat-Index within these phases, the vertical position of the tip of the C7 spinous process was derived directly from the marker placed over the C7 spinous process, whereas the vertical position of the hip joint center was approximated using the Plug-in Gait lower limb model [10] and the software Nexus (version 2.10.3; Vicon, Oxford, UK). For both movements, the index was calculated for the full duration of the lifting up and putting down phases, time-normalized to 101 data points and averaged over the five repetitions. Mean values and standard deviations were calculated to indicate typical values that can be expected in a healthy pain-free adult population.

To test the ability of the index for distinguishing between a full squat and a full stoop movement, continuous index data were compared between the two movements using paired t-tests, implemented by the MATLAB-based software package for one-dimensional Statistical Parametric Mapping (SPM; spm1d-package, www.spm1d.org) [11]. In addition, to investigate possible associations between the continuous index data and body height as well as upper vs. lower body proportion, SPM-based linear regression analyses were conducted. Statistical significance for all tests was accepted at the *p* ≤ 0.05 level.

## Results

The calculations resulted in indices of lower than 50 and higher than 80 for the squat and stoop movements, respectively, during the first half of the lifting-up and the second half of the putting-down phases (Fig. 2, top row). The mean indices for these phases were lower than 30 and higher than 90 for the squat and stoop movements, respectively. During the second half of the lifting-up and the first half of the putting-down phases, indices for the squat movement tended to increase towards 50, whereby indices for the stoop movement remained relatively consistent. It should be noted, however, that the indices of 2 participants during lifting-up and 1 participant during putting-down with the stoop technique dropped down to zero within the last and first 10% of the lifting phase, respectively.

**Fig. 3:**
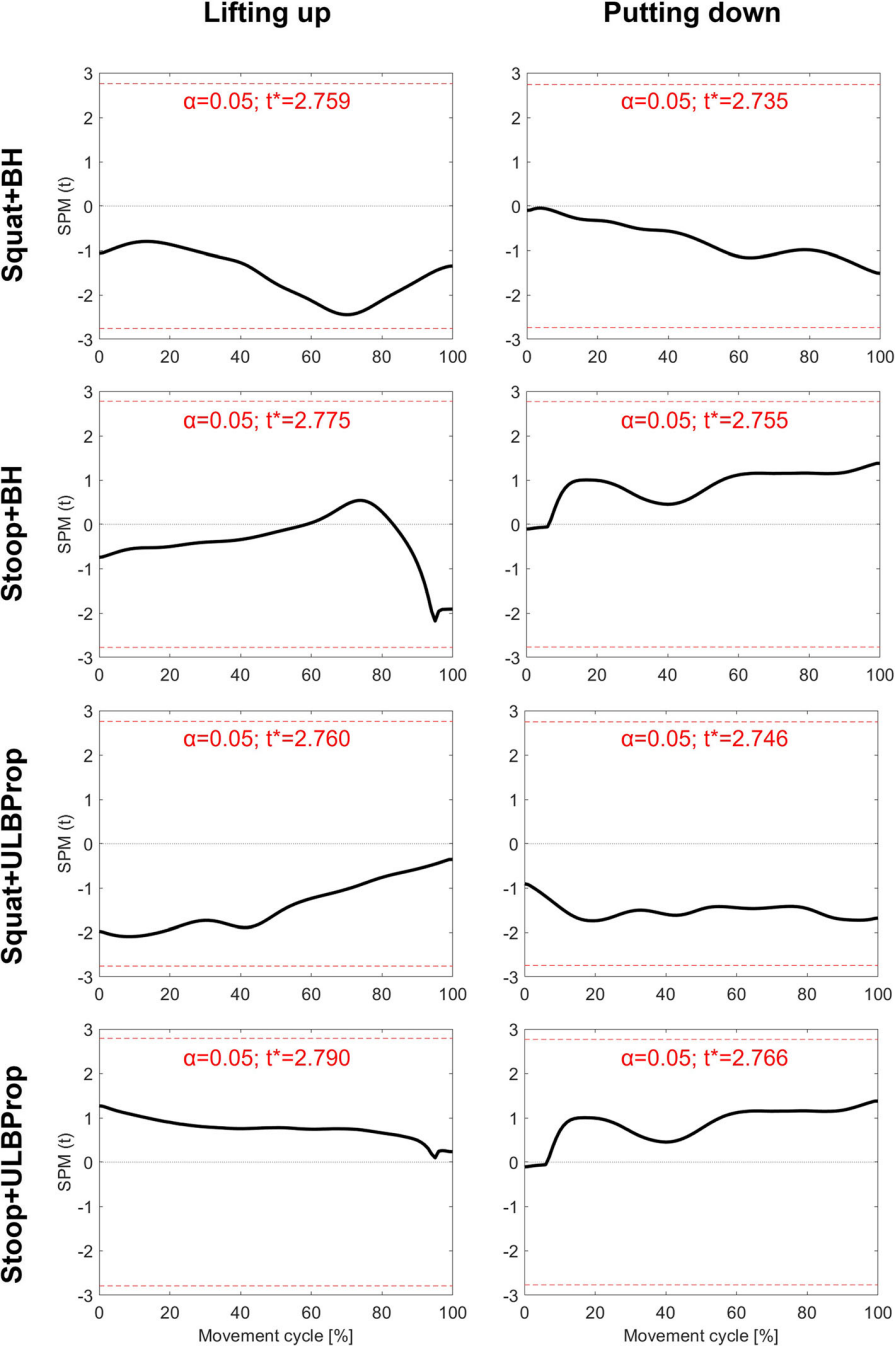
Correlations between the Stoop-Squat-Indices of the two lifting techniques and body height (BH) as well as upper vs. lower body proportion (ULBProp) using Statistical Parametric Mapping-based linear regression analyses

The comparisons of the indices between the squat and stoop movements revealed statistically significant differences (*p* < 0.001) over the full duration of the lifting-up and putting-down phases (Fig. 2, bottom row), indicating that the index was able to distinguish between the two movements at each instance of time, even during almost upright standing. Linear regression analyses did not reveal any associations between the index and body height or upper vs. lower body proportion (Fig. 3).

## Discussion

The current study aimed at introducing a novel and simple measure for quantifying the stoop-squat behavior during object lifting and to establish normative values for healthy pain-free adults. The proposed method, the Stoop-Squat-Index, describes the proportion between trunk forward lean and lower extremity joint flexion, with possible values ranging from 0 (full squat lifting) to 100 (full stoop lifting). Normative values showed mean values of lower than 30 and higher than 90 for the most relevant phases of the squat and stoop movements, respectively. Comparisons of the normative indices between the squat and stoop movements showed statistically significant differences over the full duration of the lifting phases, whereas no associations between the index and body height or upper vs. lower body proportion were found.

The main advantages of the proposed method are that the index is simple to calculate and can be derived from uncalibrated (i.e., actual distances in meters unknown) conventional video recordings, enabling large-scale in-field measurements with relatively low expenditure. However, since the identification of the relevant landmarks (i.e., the tip of the C7 spinous process and the hip joint center) on the video images might not always be as clear as motion capture data, comparative evaluations are needed to determine the accuracy of such video-based measurements. Preliminary (unpublished) results of an ongoing validation study indicate that the measurement errors can be expected to be below 5 index points for the phases where the box is close to the floor during both a full squat and a full stoop movement. For the phases close to upright standing during a full stoop movement, on the other hand, errors might be somewhat higher. As shown in the results of the current study, indices of few participants dropped abruptly down to zero during these phases, which might be attributable to slightly more flexed knee joints during the bending over phases resulting from hamstring tightness.

The larger variation of index values for squat lifting compared to stoop lifting, especially during the phases where the box was close to the floor, might be explained by differences in ankle joint flexibility and different arm lengths. Participants with sufficient ankle dorsiflexion capability (i.e., the ones that could keep the heels continuously on the floor) and proportionally longer arms were able to perform the squat lifting maneuver with no or only minimal trunk forward lean, whereas others were required to bend their trunk noticeably more forward to reach the box or to put the box back on the floor. During stoop lifting, most participants slightly flexed their knees due to hamstring tightness. However, it seems that this had less of an impact on the index than ankle joint flexibility or arm length during squat lifting.

Due to a continuum of values that can be assigned for the movements between full squat and full stoop, this novel index is well suited for studies investigating possible causal effects between whole-body lifting strategies and LBP incidence as well as potential interactions between psychological factors and object lifting strategy. A recent study, for example, showed that a higher fear of “round-back lifting” (i.e., lifting with a flexed spine) was significantly associated with less lumbar spine flexion in healthy pain-free adults that were lifting a 5 kg-box [12]. However, since only lumbar spine flexion was evaluated, the study did not allow any conclusions on whether this reduced lumbar spine flexion also meant that the participants adopted more squat lifting behavior. For this reason, we decided to use the Stoop-Squat-Index described in the current paper to exploratively reanalyze the data and found that the fear of “round-back lifting” was actually not related to stoop-squat behavior [13]. These important findings clearly show that studies reporting altered lumbar spine flexion during lifting such as the ones meta-analyzed by Saraceni et al. [6] do not necessarily imply lifting strategy alterations on a whole-body level.

Because of its simple nature and the fact that it can be fairly easily derived from conventional videos such as recorded with a smartphone or a tablet, the Stoop-Squat-Index might even find its way into everyday clinical practice. For example, it could serve as a documentation parameter when physiotherapists use reassurance interventions such as guided stoop lifting for decreasing fear of movement in LBP patients. In such a case, it would then make sense to not calculate the index for the whole lifting maneuver but only for a specific instance in time, e.g., right after lifting the box off the floor.

A major limitation of the Stoop-Squat-Index is that it does not allow any statements on how trunk forward lean is achieved, which could be either by tilting the pelvis anteriorly while keeping the spine straight or by flexing the spine while keeping the pelvis in an upright position. For this reason, it is suggested that the index is always used in combination with spinal flexion measurements. To avoid using sophisticated methods that require a laboratory setting, this could be achieved by using portable and easy-to-apply inertial measurement unit- or strain gauge-based systems [14], which have partially already been validated for quantification of lumbar lordosis angles during object lifting [9]. Limitations of the normative data provided in this study are that they only represent relatively young and healthy adults that were lifting a box with a weight of 15 kg. The stoop-squat behavior might be different in other healthy or patient populations and when lifting lighter or heavier objects. Future studies using the Stoop-Squat-Index to investigate lifting strategies within different populations or with different weights are therefore encouraged to establish their own normative values and use the values provided in this study to support the interpretation of their findings.

## Conclusion

The proposed index represents a novel and powerful measure for evaluating stoop-squat behavior during object lifting, which can fairly easily be derived from conventional video recordings with an expected high accuracy. When used in combination with lumbar spine flexion measurements, the index can contribute important information, which is necessary for comprehensively evaluating whole-body object lifting strategies.

## Data Availability

The normative values established in this study can be obtained upon request.
